# Clinical value of radiomics and machine learning in breast ultrasound: a multicenter study for differential diagnosis of benign and malignant lesions

**DOI:** 10.1007/s00330-021-08009-2

**Published:** 2021-05-21

**Authors:** Valeria Romeo, Renato Cuocolo, Roberta Apolito, Arnaldo Stanzione, Antonio Ventimiglia, Annalisa Vitale, Francesco Verde, Antonello Accurso, Michele Amitrano, Luigi Insabato, Annarita Gencarelli, Roberta Buonocore, Maria Rosaria Argenzio, Anna Maria Cascone, Massimo Imbriaco, Simone Maurea, Arturo Brunetti

**Affiliations:** 1 Department of Advanced Biomedical Sciences, University of Naples “Federico II”, Via S. Pansini, 5, 80131 Naples, Italy; 2 Department of Clinical Medicine and Surgery, University of Naples “Federico II”, Naples, Italy; 3 Laboratory of Augmented Reality for Health Monitoring (ARHeMLab), Department of Electrical Engineering and Information Technology, University of Naples “Federico II”, Naples, Italy; 4 Department of Radiology, A.O.U. San Giovanni di Dio e Ruggi d’Aragona, Salerno, Italy

**Keywords:** Machine learning, Breast cancer, Ultrasound

## Abstract

**Objectives:**

We aimed to assess the performance of radiomics and machine learning (ML) for classification of non-cystic benign and malignant breast lesions on ultrasound images, compare ML’s accuracy with that of a breast radiologist, and verify if the radiologist’s performance is improved by using ML.

**Methods:**

Our retrospective study included patients from two institutions. A total of 135 lesions from Institution 1 were used to train and test the ML model with cross-validation. Radiomic features were extracted from manually annotated images and underwent a multistep feature selection process. Not reproducible, low variance, and highly intercorrelated features were removed from the dataset. Then, 66 lesions from Institution 2 were used as an external test set for ML and to assess the performance of a radiologist without and with the aid of ML, using McNemar’s test.

**Results:**

After feature selection, 10 of the 520 features extracted were employed to train a random forest algorithm. Its accuracy in the training set was 82% (standard deviation, SD, ± 6%), with an AUC of 0.90 (SD ± 0.06), while the performance on the test set was 82% (95% confidence intervals (CI) = 70–90%) with an AUC of 0.82 (95% CI = 0.70–0.93). It resulted in being significantly better than the baseline reference (*p* = 0.0098), but not different from the radiologist (79.4%, *p* = 0.815). The radiologist’s performance improved when using ML (80.2%), but not significantly (*p* = 0.508).

**Conclusions:**

A radiomic analysis combined with ML showed promising results to differentiate benign from malignant breast lesions on ultrasound images.

**Key Points:**

*• Machine learning showed good accuracy in discriminating benign from malignant breast lesions*

*• The machine learning classifier’s performance was comparable to that of a breast radiologist*

*• The radiologist’s accuracy improved with machine learning, but not significantly*

**Supplementary Information:**

The online version contains supplementary material available at 10.1007/s00330-021-08009-2.

## Introduction

Ultrasound (US) has gained an established role in the assessment of breast lesions, showing several indications in female subjects including cases of palpable lumps, as first diagnostic tool in patients younger than 40, and for the evaluation of suspicious findings at mammography or magnetic resonance imaging [1]. According to the Breast Imaging-Reporting and Data System (BI-RADS) risk assessment and quality assurance tool, lesions are classified into different categories reflecting malignancy probability [2]. Several BI-RADS US descriptors are provided to aid in standardizing breast lesion characterization. According to a recent meta-analysis, the pooled sensitivity and specificity of US used as primary tool in detecting breast cancer lesions are 80.1% and 88.4%, respectively [3].

Radiomics is a complex multi-step process that allows extracting quantitative data from medical images, for example using texture analysis, to build clinically useful prediction models and decision support tools [4, 5]. In oncologic patients, radiomics features can be used to non-invasively assess intratumoral heterogeneity on routinely performed imaging exams [6]. When applied to medical images, artificial intelligence (AI) techniques employing machine learning (ML) algorithms have shown valuable results in image-recognition tasks, also being able to extract quantitative parameters reflecting image heterogeneity [7]. Mainly embraced for classification tasks, different ML approaches can be considered, with the most commonly applied in radiology being supervised learning (requiring labeled input data) and unsupervised learning (requiring unlabeled input data) [8]. Furthermore, several different algorithms are currently available, from those more easily interpretable (such as decision trees) to more complicated and harder to interpret ones (such as the convolutional neural networks used in deep learning) [9]. A large variety of possible clinical applications for AI in breast imaging has been described, applied to either US, digital breast tomosynthesis, or magnetic resonance imaging, ranging from differential diagnosis of breast lesions to breast cancer molecular subtype identification and prognosis prediction [10, 11]. According to previous experiences, radiomic analyses applied to breast US images have shown a good accuracy in the differential diagnosis of BI-RADS 4 and 5 lesions as well as to discriminate triple negative breast cancer from fibroadenomas [12, 13]. Often, these investigations lack external validation as analyzed data originates from a single institution. Similarly, the added clinical value of the proposed ML tools is not always assessed, leaving some doubts on the real-world benefits of AI.

Therefore, the scope of this study was threefold: (1) to assess the accuracy of a radiomic approach paired to ML applied to US images acquired in routine clinical practice to differentiate benign from malignant BIRADS 2–5 breast lesions, with internal and external testing; (2) to compare its diagnostic accuracy with that of a dedicated breast radiologist; (3) to verify whether the performance of the radiologist could be improved by the use of the proposed ML algorithm.

## Materials and methods

### Patient population

The institutional review board approved this retrospective study and written informed consent was waived. All breast ultrasound examinations performed between November 2018 and June 2019 at the University Hospital “San Giovanni di Dio e Ruggi D’Aragona” in Salerno, Italy (Institution 1), and the Diagnostic Imaging Unit of the University of Naples “Federico II,” Italy (Institution 2). Clinical indications for performing breast US were both routine check-up and assessment of palpable breast lesions or diagnostic in-depth analysis of breast lesions detected elsewhere. US examinations were performed by two radiologists in each institution, with 8 to 20 years of experience in breast imaging. Inclusion criteria were as follows: > 18-year-old patients with at least one BI-RADS 2, 3, 4, or 5 lesion. Exclusion criteria were as follows: unavailable follow-up for BI-RADS 3 lesions, or pathological confirmation for BI-RADS 4 and 5 lesions; US images not suitable for radiomic analysis due to artifacts; BI-RADS 2 cystic lesions. Breast lesions from Institution 1 were used as the training set, while those from Institution 2 were used as an external test set, to assess the radiologist’s performance with and without the aid of AI. The standard of reference consisted of 6 months follow-up (US and/or mammography) for BI-RADS 3 lesions and pathology examination by means of Tru-Cut biopsy or surgical excision for BI-RADS 4 and 5 lesions.

### Image acquisition

US examinations were performed using a LOGIQ S8, GE Healthcare (Institution 1) and a Logos HiVision E-Hitachi (Institution 2) US scanners, employing a high-frequency linear probe with radial, transverse, and longitudinal scans on both breasts. DICOM images were recorded and stored in the respective institutional digital archives.

### Image conversion and segmentation

US examinations were evaluated by a dedicated breast radiologist (VR) with 8 years of experience in breast imaging who selected and retrieved the DICOM image of each breast lesion. 2D B-mode images in which the lesion was fully included, free from artifact and any measurements, were selected. As US images were originally encoded as three-channel RGB images, they were converted to grayscale applying an ITU-R 601-2 luma transform:

$$ \mathrm{L}=\mathrm{R}\ast 299/1000+\mathrm{G}\ast 587/1000+\mathrm{B}\ast 114/1000 $$

Where L is the luminance value, and R, G, and B the original values for the red, green, and blue channels for each pixel. This was performed with the Image module of the PILLOW Python package (v2.2.1).

**Fig. 1:**
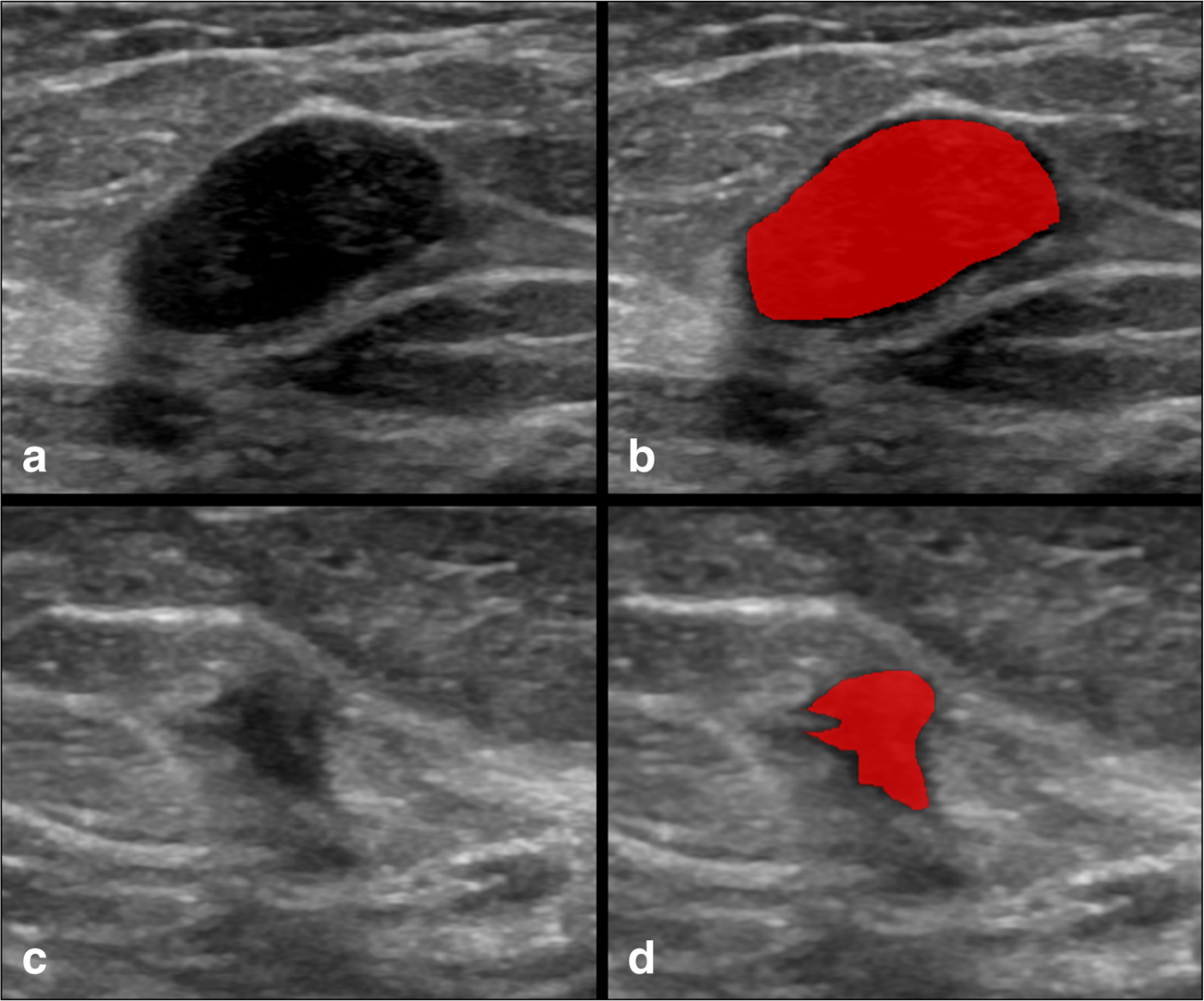
Examples of lesion annotation. The upper row (**a**, **b**) shows placement of a region of interest on a benign lesion, while **c** and **d** depict a malignant lesion before and after manual segmentation.

Subsequently, the same radiologist performed manual lesion segmentation using a dedicated software (ITKSNAP, v3.8.0) obtaining 2D regions of interest (ROIs) (Fig. 1) [14]. To assess feature stability, manual segmentation was also performed independently by two senior radiology residents (A.V. and A.V.) on 30 randomly selected patients from the training set in order to calculate feature intraclass correlation coefficient (ICC) [15].

### Image preprocessing and feature extraction

A dedicated open-source Python-based software (PyRadiomics, v2.2.0) was employed for image preprocessing and 2D radiomic feature extraction [16] [17].

As voxels were already isotropic in-plane, no resampling was necessary prior to feature extraction. On the other hand, gray-level whole-image normalization was performed to ensure comparability of images acquired on different scanners and with varying settings, with a resulting range of 0–600. As suggested by the developers, a fixed bin width (= 3) was used for discretization. Other than from the original images, features were also extracted from Laplacian of Gaussian (LoG, sigma = 1, 2, 3, 4, 5) and wavelet (all high- and low-pass filter combinations on x and y planes) filtered ones. The use of image filters can reduce image noise and highlight textural characteristics. In particular, the LoG filter performs an image smoothing operation, enhancing structural edges within the image of interest. In this setting, the sigma value specifies the desired fineness or coarseness of the resulting output (lower values produce finer images and vice versa) [18]. Wavelet decompositions represent an alternative approach to remove low signal areas from the images (i.e., image smoothing and edge detection). Using high- and low-pass filter combinations, the original image is decomposed in distinct components, expanding the original signal [19]. As the best practice for medical image analysis is not established, these alternative filtering approaches have been both included in the investigation.

In regard to feature classes, 2D shape, first order, gray level co-occurrence matrix (GLCM), gray level run length matrix, gray level size zone matrix, and gray level dependence matrix ones were extracted. In particular, as image acquisition could vary in terms of depth and zoom, only adimensional 2D shape features were included to avoid biases, in particular perimeter-surface ratio, sphericity, spherical disproportion, and elongation. For the remaining classes, all available features were calculated with the exception of GLCM sum average, as suggested by the PyRadiomics developers due to known redundancy with other GLCM parameters.

Formulas and definitions of the extracted features can be found on the official documentation (https://pyradiomics.readthedocs.io/en/latest/features.html).

### Data analysis and feature selection

Feature stability testing was performed by calculating a two-way random effect, single rater, absolute agreement ICC for each. Only features with good reproducibility (ICC value ≥ 0.75) were considered stable and included in the following steps [15, 20]. ICC calculation was performed using the R “irr” package [21]. The numbers of patients (n = 30) and readers (n = 3) as well as the ICC cutoff value were based on suggestions by recent guidelines and previous ML studies [15, 20]. A MinMax scaler with 0–1 range was fitted on the training data alone, to avoid any information leakage, and used to transform both training and test sets. Successively, non-informative features showing low variance (≤ 0.01) were excluded. Similarly, highly intercorrelated features were discarded based on the pairwise correlation matrix (r ≥ 0.8). At this point, the Synthetic Minority Over-sampling Technique (SMOTE) was employed on the training data to balance the dataset [22, 23]. In detail, SMOTE creates new instances (i.e., synthetic patients) of the minority class by interpolation of data from *k* (= 5 in our study) nearest neighbors from the original population with the same label. The process is repeated until the two classes are perfectly balanced. Finally, stratified 10-fold cross-validated recursive feature elimination (RFECV) with a Logistic Regression (LBFGS solver) estimator identified the optimal number of parameters to train the ML algorithm. These data processing steps were conducted using the pandas and scikit-learn Python packages [21, 24].

### Machine learning analysis

Given the tabular nature of the data, expected number of instances available, and previous experiences, also following the recommendations made by the scikit-learn developers, a Random Forest (RF) ensemble algorithm was selected for this classification task.

Algorithm performance during the random search tuning process was assessed on the training set through 5-fold stratified cross-validation. This approach is more robust than a single train-test split and can be expected to give a better estimation of generalizability [25]. In stratified cross-validation, each of the folds the data is split preserves the class balance and is used as a validation set for an algorithm trained on the remaining (n = 4) data folds. Then, the final model was fitted on the whole training set and tested on the data from Institution 2. Its accuracy was also compared to a baseline reference value (no information rate, NIR) corresponding to the accuracy obtainable by classifying all lesions as belonging to the most frequent class (i.e., the mode of the classes). A *p* value < 0.05 was considered statistically significant. The Brier score was calculated for the model on the test set, as well as a calibration curve, to assess prediction and calibration loss of predicted probability and lesion class.

The machine learning analysis was performed using the scikit-learn Python package. Accuracy metrics were computed with the same Python package and the caret R package [21, 24].

### Radiological evaluation

A dedicated breast radiologist (R.B., 8 years of experience) from Institution 1 evaluated the same US images of the test set used for the ML analysis and classified each lesion as benign or malignant, according to the BI-RADS V edition. In detail, the radiologist assessed lesion shape, margins, orientation, echo-pattern, and posterior features assigning a score from 2 to 5. BI-RADS scores were then dichotomized as 2–3 = benign, and 4–5 = malignant for the subsequent analysis. The radiologist was blinded to patient clinical history and final diagnosis. After a 4-week washout period, the same radiologist performed a new evaluation, this time with the availability of ML predictions and probabilities for each lesion. The accuracy of the radiologist was calculated using the caret R package and also compared to the NIR baseline accuracy reference.

### Statistical analysis

Kolmogorov-Smirnov test was first performed to assess whether data were normally distributed. Accordingly, t-test and Mann-Whitney U test were performed to assess differences in terms of age and lesion size (maximum diameter) of malignant and benign breast lesions between training and test sets. Accuracy, sensitivity, specificity, and positive and negative predictive values of both ML classifier and expert radiologist were calculated. McNemar’s test was performed to assess differences in the performance between ML and the human reader and between the reader without and with the use of ML. A *p* value < 0.05 was considered statistically significant.

## Results

### Patient population

**Fig. 2:**
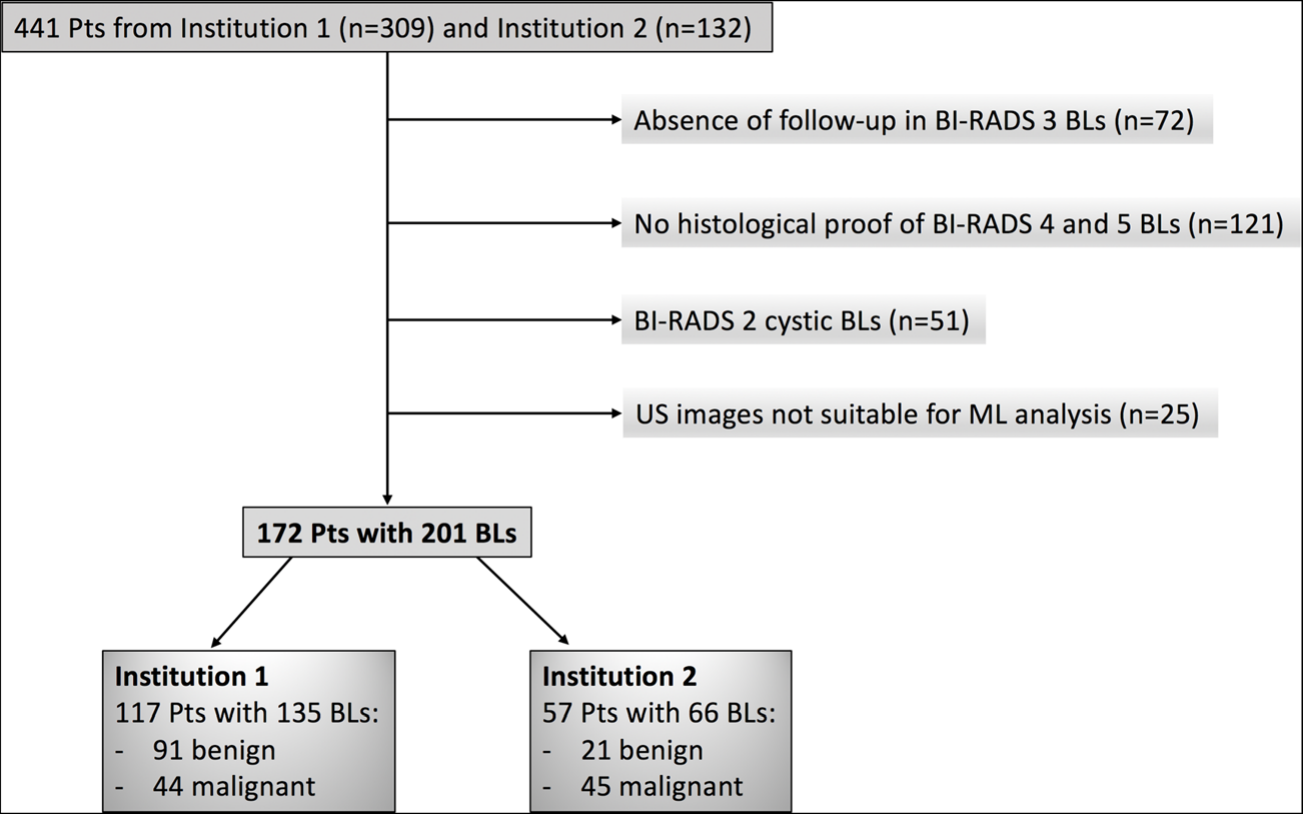
Flowchart of the patient selection process. Pts, patients; BLs, breast lesions

Based on inclusion criteria, 441 patients of which 309 were from Institution 1 and 132 from Institution 2 were reviewed. Applying our exclusion criteria, a final population of 117 patients from Institution 1 (mean age 48 years, range 15–94 years), with 135 lesions (91 benign and 44 malignant), and 57 patients from Institution 2 (mean age 52 years, range 12–85 years), with 66 lesions (21 benign and 45 malignant), was therefore included. The flowchart of patient selection process is illustrated in Fig. 2. Age was not statistically different between training and test sets at Student’s t-test (*p* = 0.177).

Mean size of breast lesions from Institution 1 was 13 mm (range 4–44 mm), while mean size of breast lesions from Institution 2 was 16.37 mm (range 4–47 mm). Training and test sets did not differ in terms of lesion size between benign and malignant breast lesions at Mann-Whitney U test (*p* values 0.794 and 0.325, respectively).

The BI-RADS assessment of the included lesions can be found in the supplementary materials as Table S1.

Fourteen BI-RADS 4 lesions were revealed as benign, while the remaining BI-RADS 4 and 2 BI-RADS 3 lesions, who showed a significant increase of lesion size at follow-up examinations, resulted in malignant pathology. Final diagnoses of histologically proven BI-RADS 3, 4, and 5 breast lesions are reported in Table 1.

**Table 1:** Diagnosis of histologically proven BI-RADS 3, 4, and 5 lesions in both training and test sets

Diagnosis	Number of lesions (%)	Training (%)	Test (%)
Malignant lesions			
Invasive ductal carcinoma	66 (75)	32 (73)	34 (76)
Invasive lobular carcinoma	10 (11)	4 (9)	6 (13)
Other*	13 (14)	8 (18)	5 (11)
Total	89	44	45
Tumor grade			
G1	15 (17)	7 (16)	8 (18)
G2	47 (53)	25 (58)	22 (49)
G3	26 (30)	11 (26)	15 (33)
Total	88	43	45
Molecular subtype			
Luminal A and B	77 (88)	38 (88)	39 (87)
HER2+	2 (2)	2 (5)	
Triple negative	9 (10)	3 (7)	6 (13)
Total	88	43	45
Benign lesions			
Fibroadenoma	6 (43)	3 (33)	3 (60)
Intraductal papilloma	4 (29)	3 (33)	1 (20)
Steatonecrosis	3 (21)	2 (22)	1 (20)
Complex cyst	1 (7)	1 (12)	
Total	14	9	5

*Intraductal papillary carcinoma, mucinous carcinoma, adenoid-cystic carcinoma; Hodgkin lymphoma, ductal carcinoma in situ

### Feature extraction, data analysis, and feature selection

A total of 520 features were extracted. Of these, 198 resulted unstable after ICC assessment and were discarded, with 322 features left. Then, 10 low variance parameters were also excluded as well as 278 highly intercorrelated ones, as resulted from the pairwise correlation matrix shown in Figure S1. After class balancing with SMOTE, from the remaining 34 features, RFECV identified a subset of 10 (Figure S2), including “original shape2D PerimeterSurfaceRatio”; “original shape2D Elongation”; “original glcm Autocorrelation”; “original gldm DependenceNonUniformityNormalized”; “log-sigma-1-0-mm-3D glcm Imc2”; “log-sigma-2-0-mm-3D glcm Correlation”; “log-sigma-3-0-mm-3D glrlm GrayLevelNonUniformityNormalized”; “log-sigma-4-0-mm-3D glcm Imc1”; “wavelet-H glcm Imc2”; “wavelet-H glrlm GrayLevelNonUniformityNormalized.” The feature selection process is summarized in Figure S3.

### Machine learning analysis

The RF hyperparameters were set as follows: bootstrap = true, class weight = none, criterion = Gini, maximum depth = none, maximum features = 5, maximum leaf nodes = none, minimum impurity decrease = 0.0, minimum impurity split = none, minimum samples leaf = 1, minimum samples split = 2, minimum weight fraction leaf = 0.0, number of estimators = 400.

In the training set, RF obtained an overall mean accuracy of 82% (standard deviation, SD, ± 6%) and positive predictive value (PPV), sensitivity, specificity, and AUC for malignant lesions respectively of 78% (SD ± 5%), 89% (SD ± 7%), 75% (SD ± 5%), and 0.90 (SD ± 0.06).

**Fig. 3:**
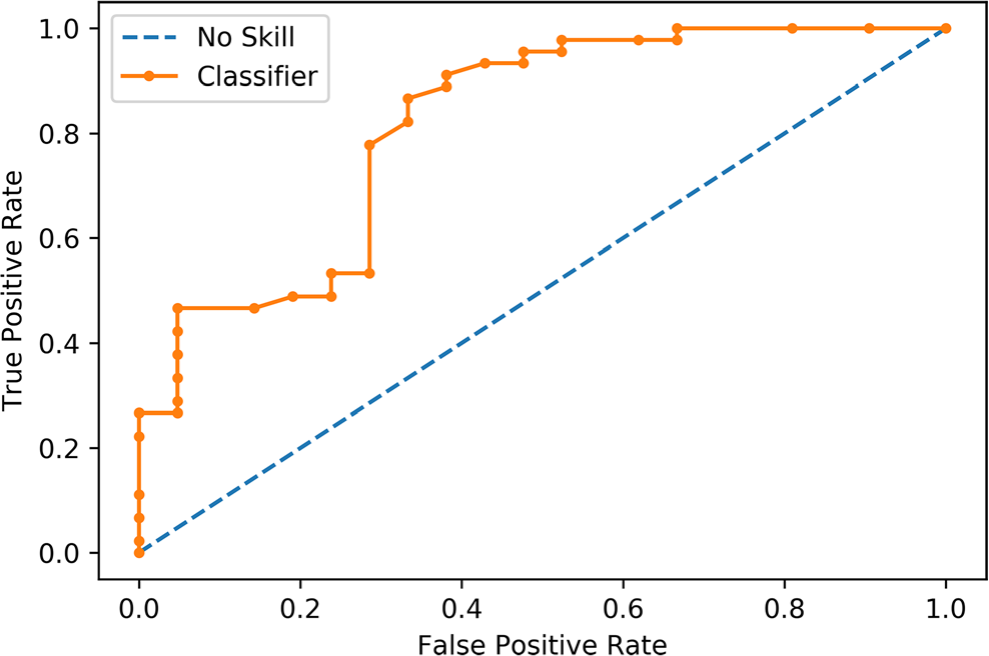
Receiver operating characteristic curve of the machine learning classifier for distinguishing benign and malignant lesions in the test set

**Fig. 4:**
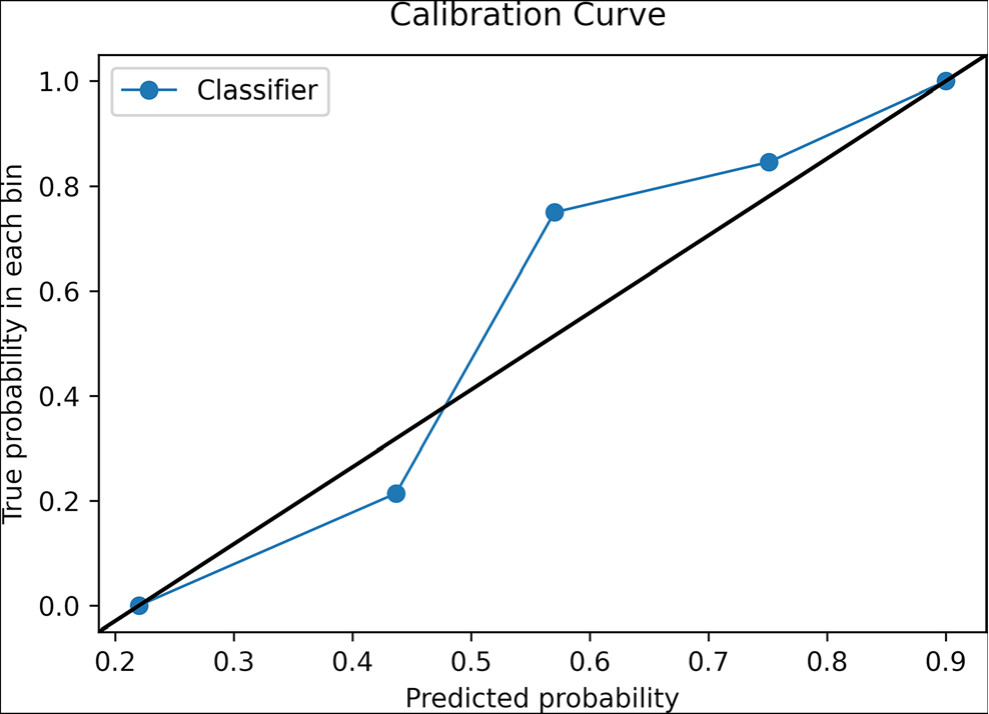
Calibration curve plot of the model in the test set. Average predicted probability is represented in the *x*-axis while the proportion of malignant lesions in the *y*-axis

In the test set, ML accuracy also was 82% (95% confidence intervals (CI) = 70–90%) with a PPV and negative predictive value (NPV) of 82% (95% CI = 74 to 89%) and 80% (95% CI = 56–93%), sensitivity of 93% (95% CI = 82–99%), and specificity of 57% (95% CI = 34–78%) for malignant lesions. RF’s accuracy results are significantly better than the NIR (*p* = 0.0098). The AUC was 0.82 (95% CI = 0.70–0.93) (Fig. 3). Regarding prediction and calibration loss on the test set, the Brier score was 0.17 and Fig. 4 presents the calibration curve plot.

### Radiological evaluation

**Fig. 5:**
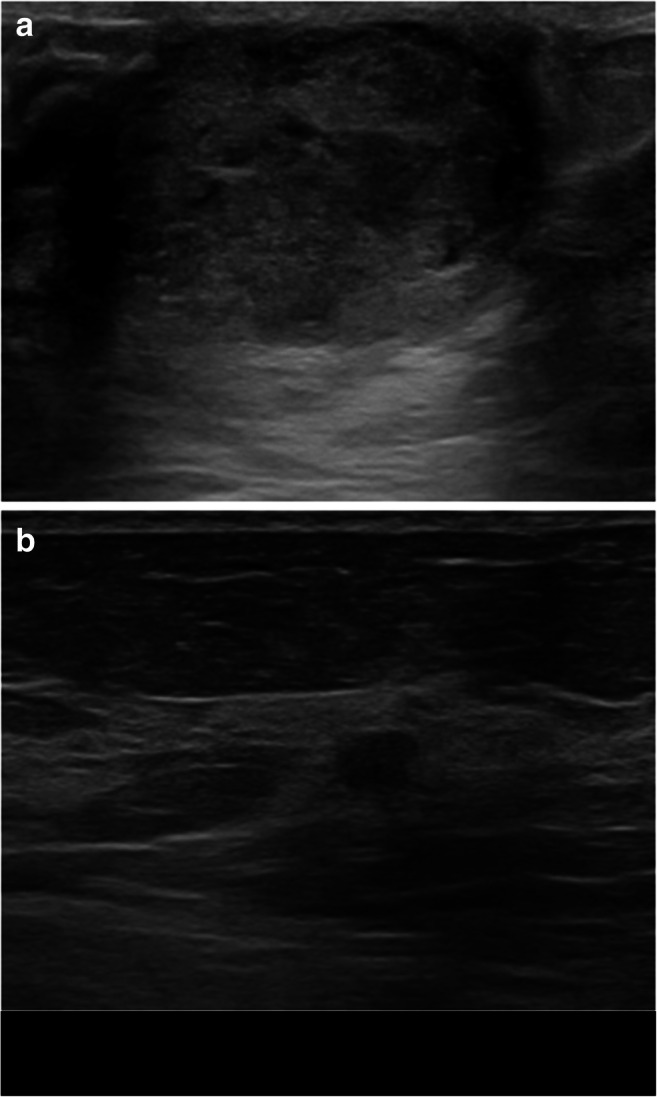
B-mode US images of a benign (**a**) and malignant (**b**) breast lesion initially misclassified by the expert radiologist and correctly diagnosed with the availability of ML reading. **a** A case of a 13-year-old patient with a 4-cm oval breast lesion with circumscribed margins but heterogeneous echo-pattern, proved to be a sclerosing papilloma after surgical excision. **b** A case of a 59-year-old patient with a 5-mm oval, hypoechoic breast lesion with circumscribed margins, histologically proved as Luminal A, G1, ductal invasive carcinoma

The expert radiologist obtained an accuracy of 79.4% (95% CI = 67–91%) on the test set, not significantly different from ML reading at McNemar’s test (*p* = 0.815). Sensitivity and specificity in identifying malignant breast lesions were 77.8% (95% CI = 62.9 to 88.8%) and 81% (95% CI = 58.1 to 94.6%), respectively, while PPV was 89.7% (95% CI = 78.1 to 95.5%) and NPV 63% (95% CI = 48.7 to 75.3%). With the availability of ML predictions, these metrics improved as follows: accuracy = 80.2% (95% CI = 67–93%), sensitivity = 88.9% (95% CI = 75.6 to 96.3%), specificity = 71.4% (95% CI = 47.8 to 88.7%), PPV = 87% (95% CI = 77.1 to 93%), and NPV = 75% (95% CI = 55.7 to 87.7%). The McNemar test comparing the ML and expert radiologist’s readings was not significantly different (*p* = 0.508). Classification tables of the comparison between the performance of the expert radiologist and ML as well as between the expert radiologist without and with the support of ML are reported in Tables S2 and S3, respectively. Examples of cases in which the expert radiologist was aided by the ML algorithm in correctly classifying benign and malignant lesions are illustrated in Fig. 5. Accuracy metrics of ML and expert radiologist without and with the availability of ML prediction are summarized in Table 2.

**Table 2:** Accuracy metrics (95% confidence interval) of ML classifier and expert radiologist without and with the availability of ML reading

	Accuracy	Sensitivity	Specificity	PPV	NPV	TP	FN	TN	FP
ML classifier	82 (70 – 90)	93 (82 – 99)	57 (34 – 78)	82 (74 – 89)	80 (56 – 93)	42	3	12	9
Expert reader	79.4 (67 – 91)	77.8 (62.9 – 88.8)	81 (58.1 – 94.6)	89.7 (78.1 – 95.5)	63 (48.7 – 75.3)	35	10	17	4
Expert reader with ML readings	80.2 (67 – 93)	88.9 (75.6 – 96.3)	71.4 (47.8 – 88.7)	87 (77.1 – 93)	75 (55.7 – 87.7)	40	5	15	6

*ML* machine learning, *PPV* positive predictive value, *NPV* negative predictive value. Data are expressed as percentages

## Discussion

In this study, we built a radiomic-based ML model to differentiate benign from malignant breast lesions on US. The resulting RF algorithm obtained an accuracy of 82%, with high sensitivity (93%) but low specificity (57%) in classifying benign and malignant breast lesions. It performed significantly better than the baseline NIR (*p* = 0.0098) and showed higher accuracy compared to the expert radiologist (82% vs 79.4%), even if this difference did not reach statistical significance (*p* = 0.815). Even though the radiologist’s accuracy increased to 80.2% with the aid of ML predictions, this difference also proved not statistically significant (*p* = 0.508).

As with all ML studies, our results should be interpreted by taking into account the data that was employed to build and test the model. We wished to focus on challenging lesions, excluding all cystic lesions which do not pose a real diagnostic challenge. This design choice is also reflected in the performance of the breast radiologist, slightly lower than what could be expected from the literature [3, 26]. It should be acknowledged that reviewing US images is not the same as performing a complete examination, but this could not be avoided due to the study’s retrospective nature. In this setting, RF outperformed both the baseline reference and the radiologist, demonstrating promising performance. The improved performance of the radiologist with the aid of ML is also suggestive of its usefulness in clinical practice. Indeed, the sensitivity of the expert radiologist raised up to 88.9% vs 77.8%, to the detriment of the specificity that, instead, decreased from 81 to 71%. The increase in terms of sensitivity is advisable as it could reduce the possibility to miss malignant lesions. It is interesting to note that the radiologist with ML still performed worse than ML alone, probably due to lack of trust in the model’s predictions.

While the model’s accuracy was stable across the cross-validation and test set assessment, in the latter we observed a reduction in specificity, compensated by increased sensitivity. It must be considered that the test set had a different proportion of malignant cases (n = 45/66, 68%) compared to the training one (44/135, 33%), and overall challenging lesions, as demonstrated by the radiologist’s performance.

Our findings support a possible clinical role for a US radiomic-ML tool in the characterization of benign and malignant breast lesions, in line with previous studies conducted using US radiomic features with [27–29] and without ML [12, 13, 30, 31]. For example, logistic regression models were developed using radiomic features extracted from US images to discriminate benign from malignant lesions [31], predict the presence of cancer in BIRADS 4 and 5 lesions [12], and differentiate fibroadenomas from triple negative breast cancer [13] with AUC values of 0.886, 0.928, and 0.834–0.864, respectively. Furthermore, images obtained from automated whole breast US were used to extract texture, shape, and ellipsoid features and also analyzed using a logistic regression model, reporting an accuracy of 85% in classifying breast masses [30]. Deep learning networks were also tested to characterize benign and malignant breast lesions, with AUC values ranging from 0.80 [28] to 0.95 [29]. ML algorithms using radiomic features based on US BIRADS lexicon were also assessed by Fleury et al [32]; the Support Vector Machine resulted as the best classifier, with an AUC value of 0.84 in characterizing breast lesions. To the best of our knowledge, this is the first study assessing the usefulness of a ML classifier using shape, first order, and texture features from both original and filtered US images to classify benign and malignant breast lesions in a challenging dataset. The possibility to implement such a tool in the routine clinical practice would have tremendous implications in the management of breast lesions, considering that US is the first-level imaging modality for their assessment. In a future perspective, it would be possible to non-invasively characterize breast lesions using a widespread imaging modality, thus reducing the recourse to breast biopsy, as well as to reserve the use of second level and more expensive imaging techniques, such as MRI, to selected cases.

Limitations of our study are represented by its retrospective nature and the relatively limited patient population. However, the multicentric study design allowed to validate the AI algorithm on an external test set from a different institution, thus determining its robustness and generalizability. As stated above, the retrospective nature of the study did not allow for a standard evaluation of patients by the radiologist, who was limited to reviewing US images. Also, the final populations from the two institutions showed different proportions of malignant lesions, also understandable in light of the retrospective design of the investigation.

In conclusion, a radiomic approach paired to ML was accurate to differentiate benign from malignant BIRADS 2–5 breast lesions on US, showing a performance comparable to that of an experienced radiologist. Further studies on a larger cohort of patients and with a prospective design are necessary to confirm our promising findings.

## Supplementary Information

ESM 1 (DOCX 4037 kb)

## Abbreviations

AI: Artificial intelligence
BI-RADS: Breast Imaging-Reporting and Data System
GLCM: Gray level co-occurrence matrix
ICC: Intraclass correlation coefficient
ML: Machine learning
NIR: No information rate
NPV: Negative predictive value
PPV: Positive predictive value
RF: Forest Ensemble Algorithm
RFECV: Cross Validated Recursive Feature Elimination
ROIs: Regions of interest
SD: Standard deviation
SMOTE: Synthetic Minority Oversampling Technique
US: Ultrasound

## Declarations

### Guarantor

The scientific guarantor of this publication is Professor Arturo Brunetti.

### Statistics and biometry

No complex statistical methods were necessary for this paper.

### Methodology

• Retrospective

• Observational

• Multicenter study
