# Supplementary material for: Clinical value of radiomics and machine learning in breast ultrasound: a multicenter study for differential diagnosis of benign and malignant lesions
Source: Eur Radiol. 2021 May 21;31(12):9511–9. doi: 10.1007/s00330-021-08009-2 (PMC8589755; doi:10.1007/s00330-021-08009-2)
Supplement: Supplementary file 1 — (DOCX 4037 kb) [file 330_2021_8009_MOESM1_ESM.docx]

**SUPPLEMENTARY MATERIALS**

**Table S1.** BI-RADS assessment of the included breast lesions from Institution 1 and 2.

| **BI-RADS Assessment** | **Institution 1** | **Institution 2** | **Standard of reference** |
| --- | --- | --- | --- |
| 2 | 59 | 2 | Follow-up |
| 3 | 27 | 12 | Follow-up |
| 4 | 15 | 24 | Pathology |
| 5 | 34 | 28 | Pathology |
| Total | 135 | 66 |  |

**Table S2**. 2x2 classification table of the comparison between the performance of the ML algorithm and the expert radiologist in classifying benign and malignant breast lesions on the test set using the Mc Nemar test (p=0.815)

|  | | **Expert Radiologist** | | **Total** |
| --- | --- | --- | --- | --- |
|  |  | **Correctly classified** | **Incorrectly classified** |  |
| **ML** | **Correctly classified** | 44 | 10 | 54 |
|  | **Incorrectly classified** | 8 | 4 | 12 |
|  | **Total** | 52 | 14 | 66 |

**Table S3**. 2x2 classification table of the comparison between the performance of the expert radiologist without and with the availability of ML predictions.

|  | | **Expert Radiologist + ML** | | **Total** |
| --- | --- | --- | --- | --- |
|  |  | **Correctly classified** | **Incorrectly classified** |  |
| **ML** | **Correctly classified** | 49 | 3 | 52 |
|  | **Incorrectly classified** | 6 | 8 | 14 |
|  | **Total** | 55 | 11 | 66 |

**
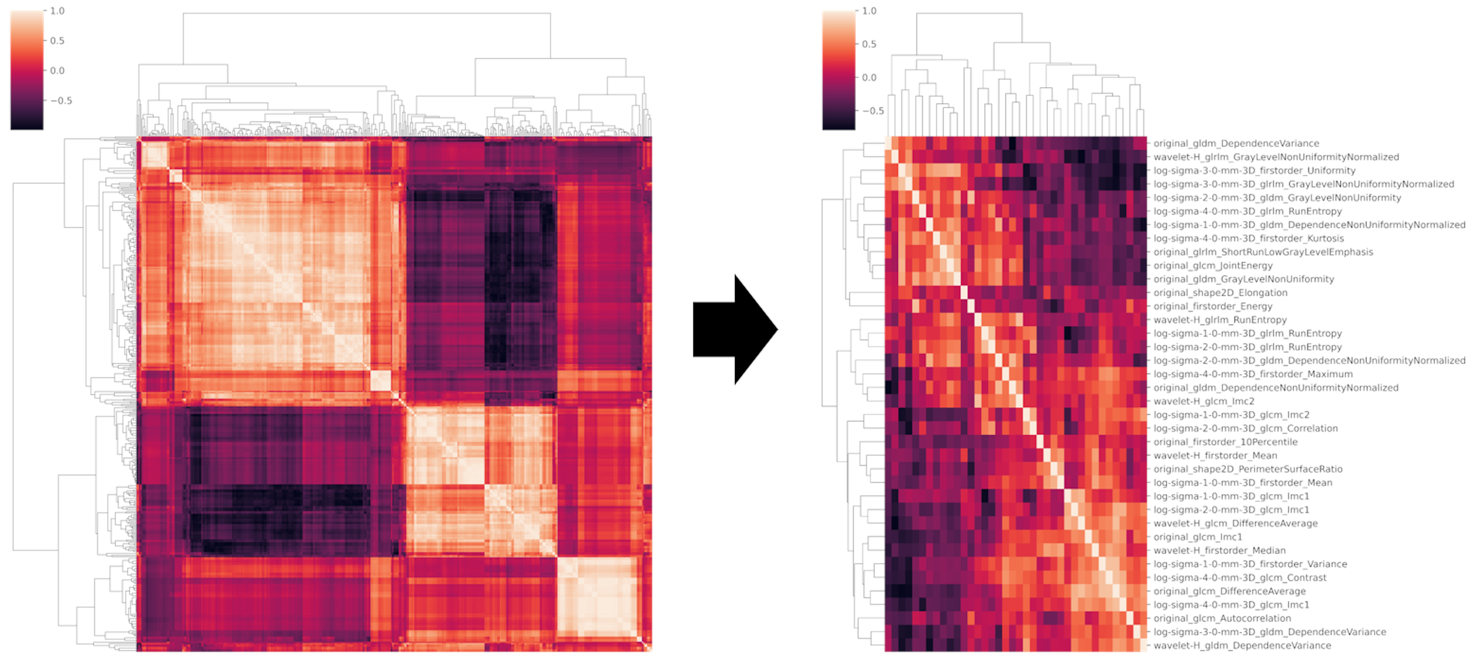
**

**Figure S1.** Clustered heatmap showing the pairwise correlation matrix of the radiomic data in the training set prior (left) and after (right) removal of highly intercorrelated features from the dataset.

**
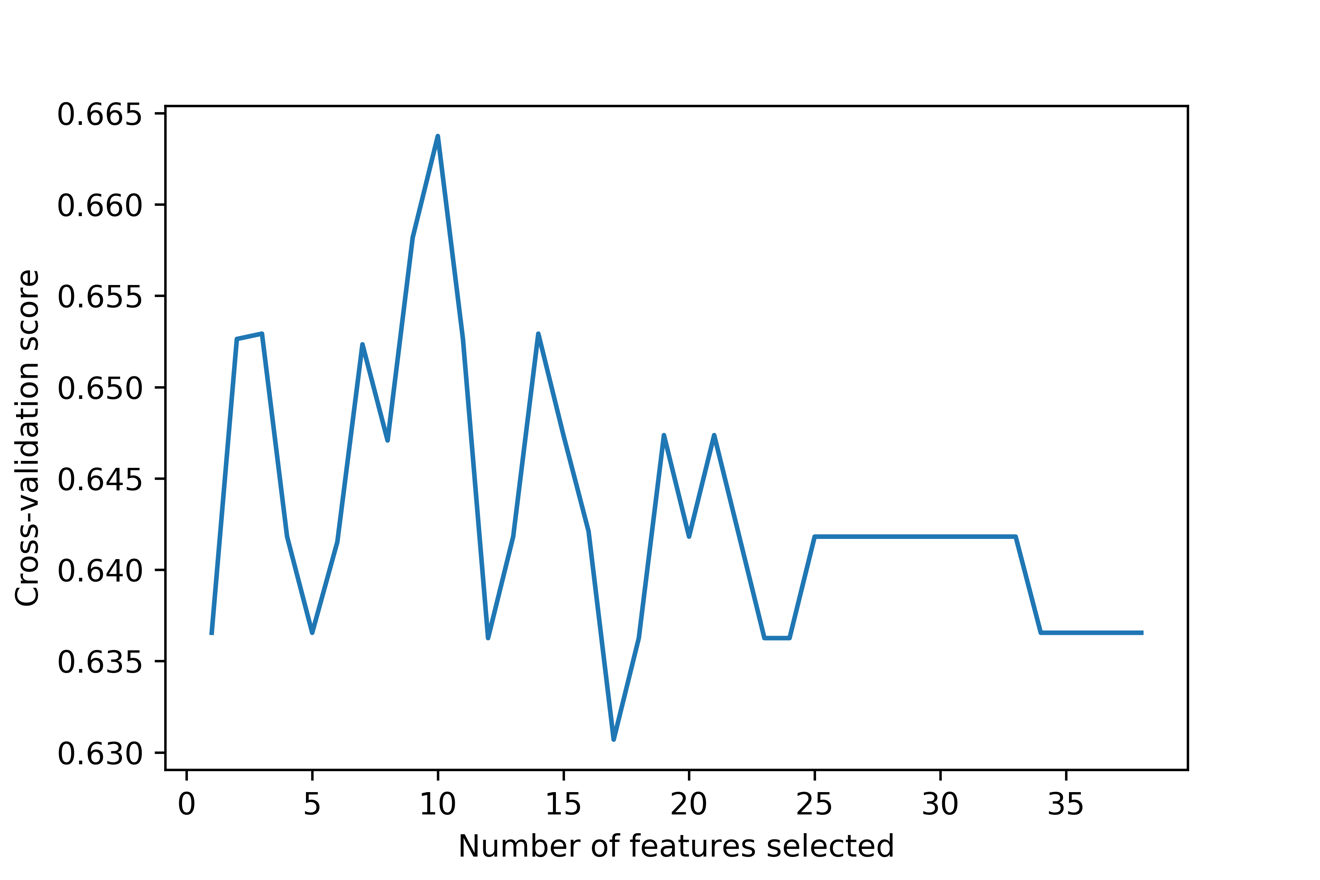
**

**Figure S2.** Lineplot presenting the results of the recursive feature elimination process. The best performance is achieved when the number of features is 10.

**
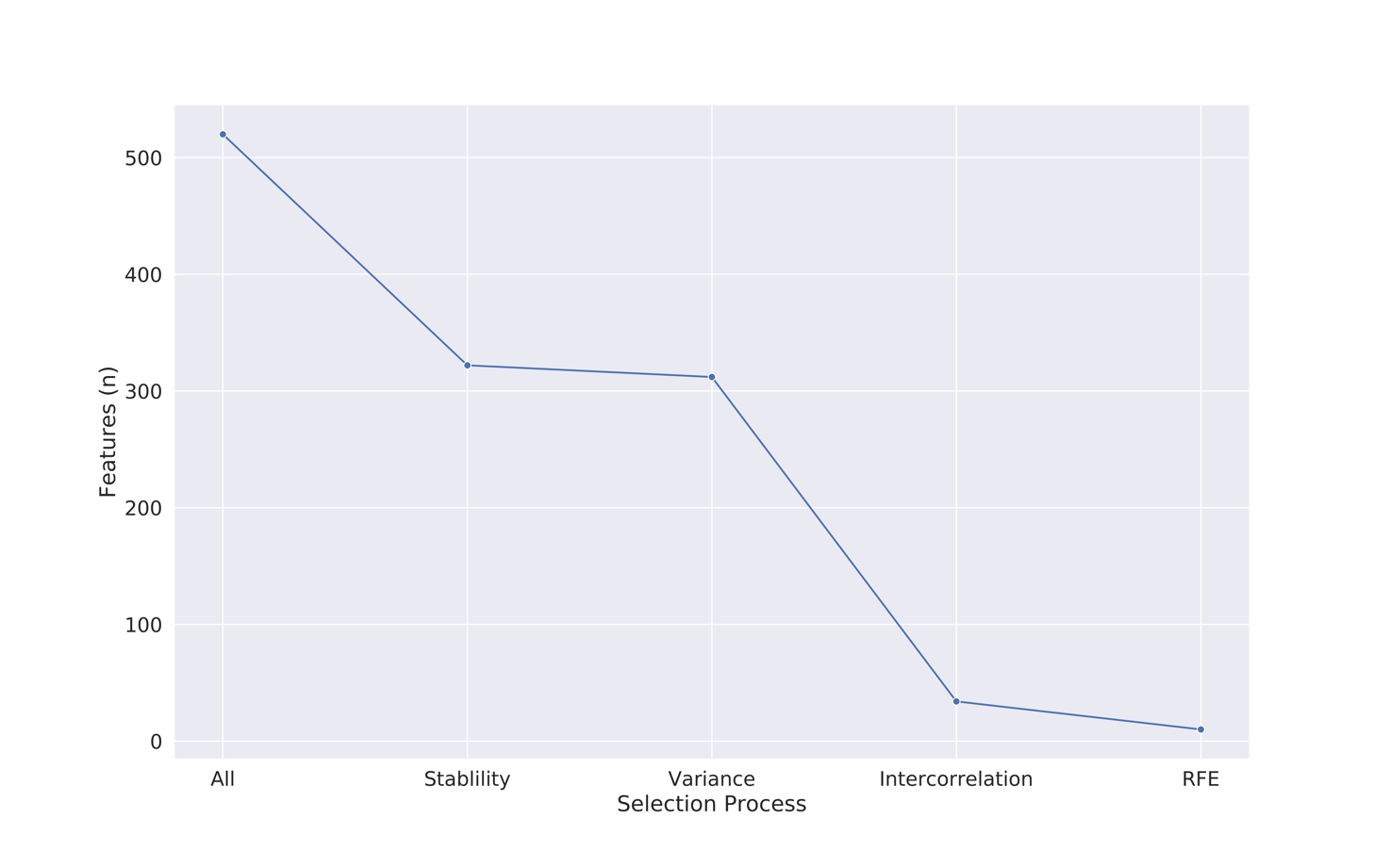
**

**Figure S3.** Lineplot summarizing the results of the feature selection process, reducing the overall number of features from a total of 522 to 10.
